# Socio-anthropological methods to study the feasibility and acceptability of the minimally invasive autopsy from the perspective of local communities: lessons learnt from a large multi-centre study

**DOI:** 10.1080/16549716.2018.1559496

**Published:** 2019-02-04

**Authors:** Maria Maixenchs, Rui Anselmo, Guillermo Martínez Pérez, Kelvin Oruko, Selidji Todagbe Agnandji, Pamela Catherine Angoissa Minsoko, Kounandji Diarra, Mahamane Djiteye, Zulfiqar A. Bhutta, Shujaat Zaidi, Carla Carrilho, Ariadna Sanz, Jaume Ordi, Clara Menendez, Quique Bassat, Khatia Munguambe

**Affiliations:** a ISGlobal, Hospital Clinic, Universitat de Barcelona, Barcelona, Spain; b Centro de Investigação em Saúde de Manhiça, Maputo, Mozambique; c Kenya Medical Research Institute, Centre for Global Health Research, Kisumu, Kenya; d Kenya Medical Training College, Nairobi, Kenya; e Centre de Recherches Médicales de Lambaréné (CERMEL), Albert Schweitzer Hospital, Lambaréné, Gabon; f Institut fϋr Tropenmedizin, Universitätsklinikum Tϋbingen, Tϋbingen, Germany; g Centre pour le Développement des Vaccins (CVD-Mali), Bamako, Mali; h Centre of Excellence in Women and Child Health, Aga Khan University, Karachi, Pakistan; i Department of Pathology, Maputo Central Hospital, Maputo, Mozambique; j Faculty of Medicine, Eduardo Mondlane University, Maputo, Mozambique; k Department of Pathology, Hospital Clinic of Barcelona, Universitat de Barcelona, Barcelona, Spain; l Consorcio de Investigación Biomédica en Red de Epidemiología y Salud Pública (CIBERESP), Madrid, Spain; m Catalan Institution for Research and Advanced Studies (ICREA), Barcelona, Spain; n Paediatric Infectious Diseases Unit, Paediatrics Department, Hospital Sant Joan de Déu (University of Barcelona), Barcelona, Spain

**Keywords:** Minimally invasive autopsy, feasibility, acceptability, methodology, ethnography, grounded theory, framework analysis, qualitative

## Abstract

The minimally invasive autopsy (MIA), an innovative approach for obtaining post-mortem samples of key organs, is increasingly being recognized as a robust methodology for cause of death (CoD) investigation, albeit so far limited to pilot studies and research projects. A better understanding of the real causes of death in middle- and low-income countries, where underlying causes of death are seldom determined, would allow improved health planning, more targeted prioritization of available resources and the implementation of coherent public health policies. This paper discusses lessons learnt from the implementation of a Feasibility and Acceptability (F&A) study evaluating the MIA approach in five countries: Gabon, Kenya, Mali, Mozambique and Pakistan. This article reports the methodological choices made to document sociocultural and religious norms around death, to examine community and relatives’ attitudes and perceptions towards MIA, and to identify factors motivating the MIA’s acceptance and refusal. We used ethnography, grounded theory and framework method approaches. In-depth and semi-structured interviews and focus group discussions with key informants, including next of kin of deceased individuals and healthcare providers, were conducted. Participant observation and direct observation of procedures and ceremonies around death were organized in all study sites. In Mozambique, MIA procedures were observed and case studies conducted. The implementation of this F&A protocol has provided critical lessons that could facilitate the future implementation of post-mortem procedures for CoD investigation. These include the need for early community engagement, staff training and preparedness, flexibility to adapt the protocol, gathering qualitative data from diverse sources, and triangulation of the data. We have applied a rigorous, effective and culturally sensitive methodological approach to assess the F&A of MIA in resource-constrained settings. We strongly recommend that such an approach is applied in settings where MIAs or similar post-mortem sensitive procedures are to be introduced.

## Background

The dearth of reliable information on causes of death (CoD) in low-income countries hinders the adequate planning and resource allocation required to achieve the specific Sustainable Development Goal 3 (Ensure healthy lives and promote well-being for all at all ages) that primarily aims to halve child and maternal morbi-mortality caused by preventable infectious diseases [1,2].

Complete diagnostic autopsies (CDA), the gold standard methodology to assess CoD, are rarely performed in resource-constrained regions, due to a variety of reasons that include poor acceptability, and a generalized lack of the necessary human resources and infrastructures to conduct them [3,4]. In these settings, the World Health Organization (WHO) recommends the use of the verbal autopsy (VA) tool as an alternative method to investigate all-cause mortality in the population, particularly for these deaths that occur outside the health system [5–7]. The VA is a relatively simple and inexpensive method that can rapidly provide clear syndromic information of the CoD at a population level. Its gradual implementation in many low-income settings has significantly improved our current understanding of CoD distribution and trends, precisely in those places where CoD information was scarcer. The VA method does, however, have also important drawbacks, being poorly specific, imprecise, and thus often leading to misclassifications [8,9]. New approaches that are affordable, technically easy to implement, accurate and acceptable to the populations’ values and meanings on death and body integrity are necessary. One innovative approach could be what is known as the minimally invasive autopsy (MIA). This approach was designed and validated in the frame of the ‘Validation of the Minimally Invasive Autopsy (MIA) Tool for Cause of Death Investigation in Developing Countries’ (CaDMIA) project [10], an ambitious research protocol undertaken in six countries with the aim of validating this new tool against the CDA, and exploring its hypothetical feasibility and acceptability. The MIA is a protocolized and systematic post-mortem methodology targeting key organs (brain, lungs, heart, liver, spleen, kidneys, bone marrow, uterus) and bodily fluids (blood and cerebrospinal fluid), and aiming to provide sufficiently good quality samples for pathological and microbiological investigations so as to substitute the complete pathological autopsy. The MIA has been validated for CoD investigation in all age groups [11–14] and has been used in different settings [15,16]. CoD results derived from the use of the MIA tool could also contribute in the future to modify and improve current analytical approaches utilized for VA data interpretation.

Assessing the F&A of innovative CoD investigation approaches is key to understanding what is culturally, socially and/or religiously congruent with local sensitivities and what is field-deployable and potentially useful in determined sentinel sites for CoD surveillance. To facilitate decision-taking for the implementation of MIA, one of the CaDMIA study aims was to assess the F&A of MIA in resource-constrained settings with different cultural, religious and geographical backgrounds. To achieve this aim, a multi-country qualitative study was conducted in Gabon, Kenya, Mali, Mozambique and Pakistan. Specific objectives of this qualitative study were: (i) To document sociocultural and religious norms around death; (ii) To evaluate willingness to know the CoD; (iii) To examine communities’ and relatives’ attitudes and behaviours towards MIA; and (iv) To identify factors motivating the acceptance and refusal to perform MIA.

Previous studies on the acceptability of post-mortem procedures have generally focused on assessing, mostly in high-income countries, the acceptability of introducing new methodologies and/or sophisticated imaging techniques so as to avoid the need to conduct a full autopsy. These studies have primarily targeted direct relatives of the deceased persons, and/or health professionals [17–19]. In middle- or low-income countries, new post-mortem methods have focused on the study of specific diseases, with more targeted single-organ biopsies (i.e. brain in malaria; lung in tuberculosis) or outbreak investigations [20]. With very few exceptions, no F&A studies have been conducted. As of today, no standard methodology has been proposed specifically for this type of investigation, which we believe is necessary before any potentially sensitive methodology is introduced in a community. This article describes the methodology proposed, in the frame of CaDMIA project, to assess the F&A of MIA and to contribute to the body of knowledge on culturally sensitive approaches to take into consideration when planning to implement MIAs in resource-constrained settings.

## Methods: the CaDMIA F&A study

### Study period and locations

The CaDMIA F&A study took place in six locations from five countries, namely Gabon, Kenya, Mali, Mozambique and Pakistan. Activities initiated in March 2013 and concluded in July 2015. The study, coordinated by the Barcelona Institute for Global Health (ISGlobal), was conducted in collaboration with the Centre de Recherches Médicales de Lambaréné (Gabon), the Centre for Global Health Research in Kisumu (Kenya), the Centre pour le Développement des Vaccins, CVD-Mali (Mali), the Centro de Investigação em Saúde de Manhiça (CISM; Mozambique), the Hospital Central de Maputo (HCM; Mozambique) and the Centre of Excellence in Women and Child Health of the Aga Khan University in Karachi (Pakistan). Three locations were urban, two rural and one semi-urban. Two were in Islamic societies and four in predominantly Christian societies. CDA were routinely conducted only in one of the two Mozambican locations. (See Table 1)

**Table 1.** Site characteristics and description.

Country	Location	Population	Urban/Rural	Main religion	Main income activity	Ethnolinguistic groups	Health facilities	Post-mortem procedures
GABON	Lambaréné	70,000	Semi-urban	Christian	Farming, Fishing	Omyene, Fang, Guischira, Tsogo	District hospital (2), Healthcare centre (2), Community Clinic (10)	Verbal autopsies
KENYA	Siaya county, Kisumu	227,400	Rural	Christian	Farming, Fishing	Luo	County Referral Hospital (1), Healthcare centre (30)	Research-related CDAs^a^
MALI	Bamako	1,810,366	Urban	Muslim	Trade, Industry, Administration, Agriculture, Farming, Crafts	Bamanans, Peuhl, Sonrais, Malinké, Soninké, Sénoufos	Tertiary hospital (1), Healthcare centre (3)	Verbal autopsies, Non-routine CDAs^b^
MOZAMBIQUE	Manhiça	160,000	Rural	Christian	Farming, Industry, Informal trading	Shangaan	District hospital (1), Rural hospital (1), Healthcare centre (12)	Verbal autopsies
	Maputo	2,000,000	Urban	Christian	Trade, Industry, Administration	Shangaan	Quaternary hospital (1)	Routine CDAs, Research-related MIAs
PAKISTAN	Karachi	14,910,352	Urban	Muslim	Trade, Industry, Administration	Urdu, Sindhi, Punjabi, Pashto, Seraiki	University hospital (7), Tertiary hospital (3), numerous private health centres	Non-routine CDAs^b^

^a^Research Study on tuberculosis

^b^Forensic CDAs

A master protocol was approved by the Hospital Clínic-Health Research Ethics Committee (Barcelona, Spain), and country-adapted protocols received approval from their respective local ethics authorities.

### Study design

Anthropology was the discipline from which study design choices were drawn. Ethnography was chosen to guide community entry, participants sampling and gathering of information related to the phenomenon of death in the health facilities and in the community, including mortuary preparations, burial ceremonies, and other peri-mortem religious and social rituals. Grounded theory and framework method were the chosen methodologies to analyse the data from both a descriptive operational perspective and an interpretative socio-anthropological perspective.

Ethnography is an immersive, cumulative and collaborative research process. It helps to understand the meanings that the communities place in oral tradition, religious rites and material products before investigating more in-depth into local cosmogonies of life and death. The ethnographer interacts directly with the community members, who are aware of the expectations placed on them as ‘data providers’ [21]. This approach allows generating qualitative, quantitative, pictorial and archaeo-historical data.

Grounded theory was first proposed by the sociologists Glaser and Strauss in 1967 [22,23]. Since then, it has been enthusiastically adopted by health scientists, especially from the nursing discipline [24,25]. Grounded theory is a set of methods that enables an inductive, systematic and iterative data gathering and analysis process [26]. Grounded theory was not proposed to test hypotheses. Rather, as an approach to generate anew theories that are ‘grounded’ in original data. Constant comparative analysis, theoretical sampling and memoing are its principal techniques to ensure trustworthiness of study results [22,26,27].

The framework analytical method was originated in policy research in the 1980s [28,29]. This method has gained popularity, especially, among multidisciplinary health research teams. It generates a matrix consisting on rows (cases, interviewees), columns (codes, categories) and cells of summarized data as a structure in which the scientist can systematically insert data from the primary sources. This framework method is useful when a descriptive analysis is the primary goal of the study; when managing large data sets; and when participants’ narratives are easily reducible to predefined conceptual categories [28,30,31].

### Study population

People who best could describe the phenomenon under scrutiny were targeted. Three groups of participants were sampled: (i) Key informants: influential leaders or individuals who knew the rituals and ethnic-religious norms and requirements for death-related events and who could be engaged in future implementation; (ii) Next of kin, not necessarily legally related, who had recently suffered the passing of a relative; (iii) Institutional and traditional healthcare providers who were either involved in mortuary procedures or who interacted with relatives of deceased (Table 2). Individuals older than 18 years and willing to consent to share their perceptions and experiences related to the study phenomena were enrolled.

**Table 2.** Study participants per site.

		Final participants per country					
Group	Expected size per country	GA	KE	ML	MZ	PK	Final participants all countries
**Key informants**	**30**	**26**	**88**	**23**	**31**	**29**	**197**
**Healthcare providers**	**30**	**29**	**53**	**37**	**33**	**31**	**183**
**Next of kin who had suffered the death of a relative**	**30**	**29**	**54**	**31**	**46**	**34**	**194**
In the preceding 0–24 hours	*10*	*4*	*4*	*2*	*9*	*5*	24
In the preceding 1–7 days		*9*	*17*	*14*	*25*	*12*	77
In the preceding 30–40 days	*20*	*16*	*33*	*15*	*12*	*17*	93
**TOTAL**	**90**	**84**	**195**	**91**	**110**	**94**	**574**

GA: Gabon; KE: Kenya; ML: Mali; MZ: Mozambique; PK: Pakistan

### Sampling and recruitment procedures

The sampling strategy and sample size varied according to the data collection tool involved. Therefore, a combination of recruitment strategies was used. For interviews, the following sampling methods were used:

- Convenience sampling [29], whereby the local team members approached potential participants who were easily accessible, including:
- key informants during community gatherings;
- institutional and traditional healthcare providers, who were approached by visiting their workplaces;
- next of kin of recently deceased individuals in the health facilities;
- next of kin of recently deceased individuals in the community, through notifications by community leaders and healthcare providers.
- Purposive sampling [29] was used to complete the number of participants required for the next of kin group according to protocol definitions.
- Snowball sampling [29], whereby the social networks of the initial participants and of other study collaborators were used to complement the recruitment process of:
- social and religious leaders;
- traditional birth attendants (TBA);
- and traditional healers, who were identified from their social networks of the initial participants and of other study collaborators.

All participants for focus group discussions (FGDs) were purposively sampled. A predetermined set of characteristics dictated the composition of each FGD, according to the protocol. In Kisumu, social science team members worked together with key informants to identify each group, namely community elders and leaders, community health workers, verbal autopsy community interviewers and village reporters. In Lambaréné, the social sciences team members contacted participants from the interviews and invited them to participate in an FGD. FGDs were composed by community and religious leaders, elders and traditional healers.

Regarding observations, the sample unit was determined by the types of locations (health facilities, home of deceased persons, and cemeteries) and types of event (the act of communicating death in the health facilities, funerals). The data generated by this set of observations revealed further locations and events worth being observed (funeral homes, churches and mosques, public spaces in the community, washing and body preparation, embalming procedures, vigils, burial ceremonies, MIA and CDA procedures, verbal autopsy interviews).

All participants for the case studies were purposely sampled. When sensitive topics emerged from the interviews, observations or FGDs (i.e. ceremonies and burial procedures for neonates; norms and procedures around miscarriages), a specific interview was organised with the person/s who could provide detailed information about the phenomenon per se. To find those informants, research assistants were supported by community leaders and healthcare providers.

Expected minimum sample sizes were pre-defined in the protocol according to previous studies’ experience in reaching saturation [32,33]. Theoretical sampling [34] was used to determine the subsequent number and type of participants and events to target. To achieve this, data were constantly interrogated and compared across participants’ transcripts and observation field notes, until the characteristics of the concepts and categories were considered saturated. For instance, the number of relatives who had lost someone within the preceding week was larger (*n* = 194 in all sites) than planned (*n* = 150 in all sites) because of the need to explore in more depth some of the themes emerging from the interviews conducted with them. On the contrary, the number of key informants was lower in Gabon and Pakistan (n = 26 and n = 29 respectively) than planned (n = 30 per site) because saturation was reached early (Table 2). The final sample size for each data collection approach was determined by saturation [35].

### Team composition and training

The overall F&A study was led and coordinated by a senior social scientist (KM). At site level, the social sciences team was composed by a local social scientist who was responsible for a team of social science assistants (i.e. community liaison officers [CLO], interviewers, FGD facilitators, observers, transcribers and data coders). Prior to data collection, a preliminary training targeting all local social scientists was held in Maputo, Mozambique, in early 2013. During the training, the lead social scientist coordinated the adaptation of the master protocol to the ethics and regulatory requirements of the five countries. During the course of the study, three more meetings were organized with the local social scientists in Manhiça, Mozambique (2013), Lambaréné, Gabon (2014) and Barcelona, Spain (2015). These meetings were useful to facilitate trainings (i.e. NVivo© software use), to exchange local experiences and to refine the analysis strategy. Hands-on trainings on the study-specific procedures for the local research assistants were organized at site level.

### Community entry

Upon ethics approval of study protocols, data collection occurred contemporaneously to community entry activities. A designated CLO or equivalent per site mapped the communities, identified the authorities’ representatives and coordinated all community entry and outreach activities. Prior to sampling participants, authorization on the conduct of research activities was sought from the community leaders in all countries. In Pakistan, additional introductory meetings with government officers, higher-rank social and religious leaders, and NGOs and academia representatives were conducted. Across all sites, under the auspices of the community leaders, multiple site-specific mechanisms such as information provision at social, cultural and religious gatherings were organized to inform community members on the study purpose. Since the outset of the study, the site social sciences teams attended a total of 101 community gatherings.

Additionally, in Mozambique and Kenya, the research institutions had a Community Advisory Board (CAB). The social sciences team engaged these CABs to discuss progress with the recruitment of participants; share preliminary results; adjust the study-specific procedures wherever recommended; and receive feedback on the potential impact of the study in the community.

### Data collection methods

Data collection was conducted by the local social scientists and research assistants. Methods included in-depth interviews (IDIs), semi-structured interviews (SSIs), focus group discussions (FGDs), participant observation, informal conversations, direct non-participant observation and case studies (Table 3).

**Table 3.** Number of events per site.

Site	IDI	SSI	FGD	OBS	Case study	Total N of events
Lambaréné, GA	42	42	4	11	0	**99**
Siaya county, Kisumu, KE	63	66	6	5	0	**140**
Bamako, ML	38	53	0	23	0	**114**
Manhiça & Maputo, MZ	39	67	0	45	4	**155**
Karachi, PK	46	48	0	20	0	**114**
**Total**	**228**	**276**	**10**	**104**	**4**	**622**

IDIs were conducted with all key informants and next of kin having suffered a death in the preceding 30–40 days. Interview thematic guides were developed, pre-tested and further refined during data collection. IDIs aimed to elucidate cultural, social and religious norms around death. Participants were encouraged to give details about customary practices around the death of a person, and to describe the process of mourning and bereavement. Perceived advantages, disadvantages and consequences of post-mortem procedures, as well as factors influencing decision to accept MIA, were identified. The willingness to know the CoD of relatives and community members was thoroughly explored. Data were collected by local social sciences assistants, with specific training and in-depth knowledge of their communities. IDIs were conducted in the local or the official language and in the place of preference of the participant (i.e. at home, at the research centre’s offices, at the health facility). The average duration of IDIs was 90 minutes.

SSIs were conducted with healthcare providers and next of kin of deceased individuals within seven days of death. The social science team considered that a SSI format was more appropriate for these groups, because it was anticipated that participants would be constrained by time due to other pressing duties (i.e. corpse preparation; scheduled clinic meetings) and when the interviewer considered that a SSI format was more appropriate to build rapport with the participant. The SSI guide aimed to explore the same topics of the IDI but in a more structured manner. The SSIs were conducted by the same social sciences assistants that conducted the IDIs, and in the language and place of preference of the participant. Duration of SSIs ranged from 15 to 45 minutes.

FGDs were planned for key informants and healthcare providers when the local social sciences team considered that participants would feel more comfortable discussing the study topic in a group of peers. All FGDs were organized by the site social scientists, who posed questions with the purpose of capturing social and community insights on the study phenomena. A previous analysis of the IDIs and SSIs transcripts was useful to detect social desirability bias during the FGD. FGDs were conducted by the same local social sciences assistants, working in teams of one facilitator and a note-taker. FGD took place in community halls, health facilities and research centres and lasted around 60–90 minutes. Average size of FGD was 11 participants, but in some cases groups were adapted to local and community needs. This was the case of Kisumu where 4 informal FGDs were held with community leaders with sizes ranging from 8 to 23 participants.

Participant observation is a data collection method fathered by Malinowski in the mid-twentieth century, now recognized as the landmark of applied ethnography [36]. The research teams conducted participant observation by witnessing procedures, customs and traditions around death at community level, (i.e. vigils, funerals, religious services, verbal autopsy home visits). During these observations, the team members took the role of an outsider who takes part in the phenomenon being observed [37,38], yet observed population groups are aware that they are being part of a research activity. Participant observation aimed at exploring community members’ and relatives’ attitudes, behaviours and relationships in this context and to understand local norms around death. During participant observation, the research team also held informal conversations with selected participants. During these conversations, which were not audiotaped, filed notes were used to annotate participants’ views, comments and clarifications on the process under observation.

Direct non-participant observation was conducted in health facilities and the community to document the events and procedures around the first moments of the death (i.e. MIAs and CDA performance; washing and preparation of the body). This technique also allowed further understanding of attitudes of the people involved in handling the body and/or in taking decisions related to subsequent steps. Hospital health workers were also under observation while performing other routine activities (i.e. issuing death certificates) to determine which approaches would be the most appropriate for a future implementation of MIA. For direct non-participant observation, team members took the position of complete observers, who avoided taking any part in the actions under observation [37,38].

Case studies [39] were conducted to obtain additional data about specific rituals and procedures according to age, gender and specific contexts, that were not possible to obtain with the interviews or the observations on accounts of their sensitive nature and/or the taboos surrounding them (for instance, rituals regarding the handling of the bodies for stillbirth and neonates in Mozambique). They were highly focused and designed to explore a specific topic in greater depth.

### Data management and analysis

All IDIs, SSIs and FGDs were tape-recorded and transcribed verbatim with the exception of four informal FGDs held in Kisumu. When no permit for tape-recording was given, detailed notes were taken and a report was elaborated by the interviewer or the facilitator. Information on observations was annotated in the format of hand-written field notes, where insights from the experience of the observer regarding the event were registered [21,40] and reports were later developed within two days in order not to lose any information. All transcriptions and reports were uploaded into NVivo 10© (QSR International Pty Ltd.), and this software was used to facilitate the organization and coding of all countries’ sets of data.

Grounded theory guided the core analysis approach, which was done iteratively with data collection. Local social scientists and their respective teams read the first set of transcripts and reports in order to identify broad themes and categories related to perceptions of death and views on post-mortem procedures. This reading allowed the refinement of a generic coding tree, which consisted of an outline of themes and suggested categories to be explored during the coding process, which consisted in linking original excerpts of transcripts and reports to the themes and categories. The coding tree was flexible and evolving, throughout the coding process and further data collection. As new themes, interpretations and theories emerged from the data, site-specific variations of the generic coding tree resulted in six distinct coding trees. Coded data were also helpful to modify the sampling strategy for next stages of data collection, to define and redefine elements in the coding tree, to guide constant comparative analysis, and to inform the introduction of new probes in the data collections guides. NVivo© data sets could be remotely accessed by the lead social scientist, who worked collaboratively with the local teams to reflect on the significance of the findings and their implications for MIA implementation.

The framework method was used to complement the analysis. This involved extracting the content of the transcripts and reports, summarizing and tabulating data into a coding matrix designed in Microsoft Excel® 2010. This structure allowed qualitative data to be aggregated into two major pre-determined concepts: (i) willingness to know the CoD, and (ii) hypothetical acceptability of MIA. Data regarding these two concepts were further disaggregated into three categories: ‘willing/accepting’, ‘unwilling/non-accepting’ and ‘only under certain circumstances’.

### Ethical considerations

Acknowledging the sensitivity of the topic under investigation, thorough care was taken in ensuring that all potential participants received comprehensive information on the study, including: the potential risks and benefits to the community, so as to allow an autonomous decision to participate in the study; and that their privacy and confidentiality was safeguarded.

Participation in these F&A activities involved minimal risk. Nevertheless, social harm could derive from the ethnographic methods. Discomfort could be caused by the private nature of the questions. People could feel uncomfortable with the presence of an outsider witnessing and taking notes at moments as private as burials, corpse preparation, family decision-making processes or death-certificate issuing. To prevent social harm, a series of rules were detailed in the study-specific procedures taking into consideration the community leaders’ and local authorities’ opinions obtained during community entry activities.

All participants in the IDIs, SSIs and FGDs were invited to provide written informed consent. Only verbal informed consent was obtained in four informal FGDs in Kisumu. If illiterate, the participants were offered to thumbprint the consent forms in the presence of an impartial witness. Versions of the consent documents were prepared in both official and local languages. Permission to tape-record data collection was asked. As part of the consent process, participants were informed about the objectives of the study, and what was expected of their participation. They were assured that their participation in the study was voluntary. Participants were also told of their right not to participate if they did not want to; to refuse to answer any question that could make them uncomfortable; or to stop the interview at any time. A signed copy of all documents, which included the contact details of the research team, was provided to all participants.

The consent documents also detailed data confidentiality procedures. Confidentiality was preserved in accordance with national legislation regarding data protection. Notes and documents containing personal identifiers were only accessible to the local research staff. Researchers from sites other than the one providing the data collection had only access, via NVivo 10©, to the anonymized contents of the interviews’ translated transcripts.

## Lessons learnt and discussion

This carefully prepared F&A study laid some important methodological foundations for a careful inquiry at site level in preparation for the implementation of MIAs in low- and middle-income countries. Data generated as part of this project were gathered directly from the triangulation of at least four different data collection methods, providing a very rich material for analysis. Panel 1 summarizes some of the common findings emerging from this multi-centre study. Indeed, lessons learnt during the preparation and implementation of our study have already been translated to real-life implementation of the MIA procedure as part of other mortality surveillance projects in Mozambique and elsewhere [41].

**Panel 1.** Useful findings and lessons learnt during the implementation of the F&A multi-centre study.

**Master Protocol and general socio-anthropological methods**
If more than one site, it is recommended to use a systematic approach (Master protocol), agreed by all sites’ team, and flexible enough to include all local idiosyncrasies The mix of different qualitative data collection methods (IDI, SSI, FGD, observations, case studies) provides complementary information, and is therefore highly recommended Triangulation of the data provides validity and increases the general discernment of the major emerging themes from the study Data should be processed, transcribed, translated and coded locally, in order not to lose meanings and views
**Preparation and implementation of activities**
For a study involving the use of any given post-mortem procedures, particularly in places where such methods have seldom been used, a community entry and engagement strategy is essential Preparatory meetings with community and religious leaders and CABs may provide useful recommendations on how to conduct data collection and how to interact and behave when approaching interviewees/observations Flexibility for adapting or changing study procedures according to these recommendations is important
**Team training and preparedness**
The very sensitive nature of the procedures and circumstances around death implies an even higher need for continuous training and preparedness of staff Psychological support may be required to diminish the consequences in study staff of taking part in post-mortem procedures and burial ceremonies; and communication with grieving families The risk of observation biases derived from such psychological impact in terms of the interpretation of data collected needs to be minimized
**Choice of Informants/Stakeholders**
Targeting different kinds of informants bring complementary perspectives Building rapport with healthcare providers may be challenging, and planning a specific mobilization strategy may be necessary
**Interview planning**
An initial prudent approach to assess such a sensitive phenomenon as death is recommended, in order to minimize negative responses For next of kin, teams need effective systems to rapidly identify participants
**Interviews with next of kin of deceased individuals**
Approaching family members is challenging but feasible if done with respect Phasing in from 30–40 days interviews to 1–7 days was useful to the team Counter-intuitively, interviews with family members in the first 24 h after death were easier to conduct than those at later stages
**Observations**
Observations provided extremely valuable information, and as a result were increased in number and nature beyond what was originally planned For observations, a different set of skills is required from the study team Start with more ‘public’ observations (cemeteries, etc.) and only move to more private ones (washing of bodies, etc.) once the study staff feels comfortable A right mix of participant and direct non-participant observations is useful Biosafety measures that are culturally appropriate must be offered to field ethnographers to ensure their adequate protection against communicable diseases during peri-mortem procedures or ceremonies and cemetery events
**Feedback to the community**
A good relationship with the community implies being available for responding any doubts, and promptly providing feedback to community and families
**Initial assumptions proven wrong**
Approaching the families was less difficult than expected Refusals to participate in the study were lower than expected It was easier to speak with the next of kin of a person deceased in the preceding hours than to that of someone having died in the preceding month The term ‘Autopsy’ and its implications was better understood at the community level due to already existing traditional procedures that implied the handling of corpses (traditional autopsies and/or traditional caesarean sections) The term ‘Autopsy’ was not necessarily perceived to have negative connotations

One of the main lessons learnt is that community engagement and feedback about the study activities is crucial. The CaDMIA F&A approach proved suitable to allow the recruitment of over 500 consenting individuals willing to discuss a topic as sensitive as death and the post-mortem care of their deceased. By July 2015, when data collection concluded, only 28 individuals had refused to enrol. Such an unexpected success in terms of willingness to participate in the study was probably the result of a very careful preparatory phase, during which intensive efforts to inform, engage and involve the community were made. Extra efforts were also made to provide continuous feedback to participants, so as to maintain their engagement and ongoing support. As a result, the study design was especially effective in voicing the participants’ concerns and preferences with regards to the implementation of MIAs in their communities.

Additionally, our results highlight the importance, in a study of this nature, of ensuring staff well-being. A topic as sensitive as death might psychologically affect the study staff and can impact on their emotional health. Previous preparation is essential, and implementing support measures during the study and facilitating the necessary space and time for study personnel to share their day-to-day emotions is highly advisable. On top of that, it is also important to consider the need to implement basic and culturally acceptable biosafety measures for the staff. As an example, wearing a biomedical mask when observing body-washing procedures in a household in rural Manhiça (Mozambique) was not considered pertinent, and thus traditional cloth (capulana) was used to cover the observer’s mouth and nose instead.

During the design stage of the project, there was a dearth of qualitative literature on the use of MIA or similarly less-invasive post-mortem methods in developing countries. The methodology initially proposed was largely informed by a single study [20] assessing the acceptability of post-mortem brain needle biopsies by community and religious leaders and by relatives of deceased in five Bangladeshi communities that were affected by a Nipah virus encephalitis outbreak. Whilst not much has been published on MIA, other studies from developing countries have reported on the usefulness of VA to report CoD [42]; performance and applicability of different VA tools for routine CoD reporting [43]; and the feasibility of using VA as a study tool [44]. Other studies have explored, with the aim to inform consent procedures, relatives’ attitudes towards CDA [45–48]. Some of these studies used qualitative methodologies. No previous multi-country study on MIA, VA or CDA, to our knowledge, was framed as a study that used multiple anthropology, ethnography and nursing-originated qualitative methods, involving such a broad representation of individuals holding decision-making power on post-mortem care; and with so many options in place for data triangulation. In this regard, our study might be the first of its kind. Only recently, a study in Soweto used key informant interviews, semi-structured interviews, focus group discussions, telephonic interviews and participant observation to further understand the community acceptability of MIA in children aged under five [49].

Our approach was successfully applied in five different countries [50] with different religious, ethnic and sociocultural backgrounds, and methods proposed are intended to be reproducible in other settings [51]. Though not usually the aim of qualitative research [52], our proposal aimed at generalizability of its findings. This intention was at the basis of conducting the study in multiple and different sites, and for the panoply of complementary methodologies utilized. In situations where one aims to fathom a study topic of a sensitive nature (i.e. death) and there is a realistic possibility of incurring social harm for the study population, other less demanding methodologies, such as knowledge, attitudes and practice (KAP) surveys may fail to fully characterize the population’s meanings, values and perceptions on the study phenomenon. In this respect, investing time and resources in the careful preparation and implementation of diverse complementary qualitative methods to gather data on death and post-mortem care from representative members of the community would therefore appear as a necessary effort to ensure any successful future implementation in that setting of activities dealing with the topic under investigation.

Researchers interested in applying this methodology in future F&A studies on post-mortem CoD investigation will need to prepare in the inception phase a risk-benefit plan guided by formative research on the context-specific characteristics of the population. During the conduct of the CaDMIA F&A, as anticipated, numerous adjustments to the master protocol were made. Due to the sensitivities, silences and taboos around death, for the sake of cultural congruency, adjustments were made without affecting the rigour and credibility of the study or without altering the study objectives.

Since the colonial period, anthropologists, missionaries and travellers have been fascinated by how people grieve, mourn and cope with bereavement and by what lies beneath the symbolic use of images, substances, music and words in their mortuary customs [53–57]. Today, from a global health perspective, all these aspects remain of great interest. As recent studies in West Africa have demonstrated, understanding community members’ preferences with regards to the mortuary preparation of the bodies of those who died from Ebola was crucial to plan community awareness to improve acceptance of safe non-traditional burial practices [58,59]. The Ebola outbreak demonstrated how crucial it is to combine socio-anthropological approaches with epidemiological research to plan public health interventions. This was the approach we followed in the CaDMIA project.

Notwithstanding the effectiveness of the CaDMIA F&A approach, further work to standardize integration of qualitative components in MIA validity and accuracy studies is warranted. Such work should take into account some gaps that the present study had. Namely, the fact that the F&A was investigated theoretically as the MIAs were not yet taking place in any of the study locations, with the exception of Maputo, in Mozambique. Further F&A studies of any new post-mortem intervention warrant enquiring at various levels, including actual or potential participants and their communities as well as stakeholders representing the institutions that are likely to implement and sustain such novel interventions. While the latter have not been adressed in this manuscript, they were also part of this study.. Given the sensitive nature of the issues discussed, this study might have been prone to memory and social desirability bias, particularly among next of kin. A systematic review of studies approaching death from a gendered and health policy perspective could help compile best practices and lessons learnt from a wide range of research experiences. Additionally, further thorough consideration of procedures to approach people who have lost a relative or who participate in mortuary preparations must be done.

## Conclusions

We have applied a systematic and culturally sensitive methodological approach to assess the F&A of implementing MIA in resource-constrained regions. Our approach was executed alongside a multi-country study that tested the accuracy of MIA, and would not have been effective had we failed to consider the insights and recommendations made by the community leaders and site research teams. Flexibility to adapt the protocol to the reality on the ground, alongside provision of measures to ensure triangulation of analysis and credibility of the study results, was a strength that helped us achieve our study objectives. In addition to allowing the investigation of the willingness to know the CoD and the F&A of MIA, our approach was critical to gather information that further informed programmatic decisions for future MIA implementation. We recommend researchers to advance in the standardization of procedures to investigate how sensitivities around death can become deterrents or motivators to accept MIA and other CoD-determination methods, as such methods will surely become of increasing public health importance in the near future.

## Paper context

The Minimally Invasive Autopsy is a robust method for cause of death determination in settings where the complete diagnostic autopsy is not feasible and/or acceptable. However, before the deployment of such a surveillance tool, it appears crucial to understand the local context in order to plan a coherent and respectful utilization. We present the methodology used to assess the feasibility and acceptability of this innovative tool at the community level.
